# Dual Function of Novel Pollen Coat (Surface) Proteins: IgE-binding Capacity and Proteolytic Activity Disrupting the Airway Epithelial Barrier

**DOI:** 10.1371/journal.pone.0053337

**Published:** 2013-01-07

**Authors:** Mohamed Elfatih H. Bashir, Jason M. Ward, Matthew Cummings, Eltayeb E. Karrar, Michael Root, Abu Bekr A. Mohamed, Robert M. Naclerio, Daphne Preuss

**Affiliations:** 1 Section of Otolaryngology-Head and Neck Surgery, Department of Surgery, The University of Chicago, Chicago, Illinois, United States of America; 2 Division of the Biological Sciences, Department of Molecular Genetics and Cell Biology, The University of Chicago, Chicago, Illinois, United States of America; 3 Monsanto Company, St. Louis, Missouri, United States of America; 4 Chromatin, Inc., Chicago, Illinois, United States of America; University Hospital Freiburg, Germany

## Abstract

**Background:**

The pollen coat is the first structure of the pollen to encounter the mucosal immune system upon inhalation. Prior characterizations of pollen allergens have focused on water-soluble, cytoplasmic proteins, but have overlooked much of the extracellular pollen coat. Due to washing with organic solvents when prepared, these pollen coat proteins are typically absent from commercial standardized allergenic extracts (i.e., “de-fatted”), and, as a result, their involvement in allergy has not been explored.

**Methodology/Principal Findings:**

Using a unique approach to search for pollen allergenic proteins residing in the pollen coat, we employed transmission electron microscopy (TEM) to assess the impact of organic solvents on the structural integrity of the pollen coat. TEM results indicated that de-fatting of *Cynodon dactylon* (Bermuda grass) pollen (BGP) by use of organic solvents altered the structural integrity of the pollen coat. The novel IgE-binding proteins of the BGP coat include a cysteine protease (CP) and endoxylanase (EXY). The full-length cDNA that encodes the novel IgE-reactive CP was cloned from floral RNA. The EXY and CP were purified to homogeneity and tested for IgE reactivity. The CP from the BGP coat increased the permeability of human airway epithelial cells, caused a clear concentration-dependent detachment of cells, and damaged their barrier integrity.

**Conclusions/Significance:**

Using an immunoproteomics approach, novel allergenic proteins of the BGP coat were identified. These proteins represent a class of novel dual-function proteins residing on the coat of the pollen grain that have IgE-binding capacity and proteolytic activity, which disrupts the integrity of the airway epithelial barrier. The identification of pollen coat allergens might explain the IgE-negative response to available skin-prick-testing proteins in patients who have positive symptoms. Further study of the role of these pollen coat proteins in allergic responses is warranted and could potentially lead to the development of improved diagnostic and therapeutic tools.

## Introduction

Allergy is the fifth leading chronic condition among Americans [1], [2]. Allergic rhinitis is acknowledged to be a major risk factor for allergic asthma. In 2004, allergic diseases resulted in 3.5 million lost workdays and 2 million lost schooldays [3]. These diseases cause a substantial health-care cost burden, approximately 12 billion dollars annually [3]. Pollen grains are among the most important allergenic triggers. Although several pollen allergens have been characterized at a molecular and structural level [4], [5], the pollen extracellular coat matrix has largely been overlooked as a source of potential allergens.

Pollen grain is synthesized inside the microsporangium in the anther of plants, where diploid mother cells undergo meiosis to produce microspores, which mature to form pollen grains. Thus, pollen grains are a critical component of plant reproduction [6]. The central cytoplasm of mature pollen is surrounded by a complex structure, consisting of an internal cellulose layer (intine), a tough and often elaborately sculptured outer wall (exine), and the pollen coat or surface [6]. The pollen-grain coat is composed of a complex set of lipids, pigments, and aromatic compounds that fill the cavities of the pollen exine; and proteins and proteolytic enzymes are necessary in the reproduction of higher plants. The cellulose-rich intine and the cytoplasmic contents are synthesized by the pollen grain itself. Unlike the intine, the pollen coat and exine are synthesized and assembled onto the pollen grain by a floral cell layer called the tapetum, which surrounds the pollen grain during its development [7], [8]. The cells of the tapetum are responsible for synthesizing and assembling the unique proteins and lipids that are on the pollen coat (surface).

To date, virtually all efforts to identify pollen allergens have focused on screening expression libraries derived from mature pollen cDNAs [9], [10], but have overlooked much of the extracellular pollen coat, a region where additional allergens may reside. The messages needed for the synthesis of the inner pollen wall, organelles, and cytoplasm are expressed in the haploid vegetative pollen nucleus. However, cDNA libraries produced from mature pollen are deficient in genes that encode the pollen coat (surface) proteins, because these proteins are synthesized and assembled onto the pollen grain by the tapetum cells of the maternal flower [7], [8]. Approximately half of the plant genome is expressed in pollen, and ∼5% is pollen-specific [11], [12]. Only a few of these transcripts overlap with those needed for assembling of the lipids, proteins, and wall material on the pollen coat (surface). Consequently, it is not surprising that most allergens identified from pollen cDNA libraries are located within the pollen cytoplasm [13].

Another reason allergens from the pollen coat are under-recognized is that currently standardized pollen allergenic extracts are prepared from defatted pollen grains (i.e., washed with organic solvents) for therapeutic immunotherapy and diagnostic skin-prick testing. The FDA requires the production of standardized extracts from defatted pollen grains with a defined level of potency to narrow lot-to-lot variability [14]. Washing of pollen with organic solvents, such as cyclohexane, has been shown to remove the pollen surface proteins [15], [16]. Although some pollen surface proteins may remain in commercial preparations, these proteins have limited solubility in the aqueous buffers used in the preparation of pollen allergenic extracts [17]. Therefore, the standardized allergenic extracts may lack important pollen coat allergens, or, at best, are unlikely to include pollen coat materials or proteins with hydrophobic domains [18]. Traditional allergen-screening methods for pollen allergens have relied on cDNAs expressed in the pollen cytoplasm, or allergen extracts from commercial sources; consequently, they have overlooked the proteins of the pollen surface.

The pollen grains of the grass of the *Poaceae* family include more than 120 plant species that cause severe to mild allergy [19]. Bermuda grass *(Cynodon dactylon),* Timothy grass *(Phleum pratense)*, Kentucky blue grass *(Poa pratensis),* and orchard grass *(Dactylis glomerata)* are some of the most potent and clinically important allergens that belong to this family. To date, four Bermuda grass pollen (BGP) allergens (all cytoplasmic) have been identified and cloned: 1) Cyn d 1, a ß-expansin; 2) Cyn d 7, a calcium-binding protein; 3) Cyn d 12, a profiline; and 4) Cyn d 24, a pathogenesis-related protein [20], [21], [22], [23]. Cyn d 1 is a major Bermuda grass allergen [20]. Although several allergens have been identified, multiple reports suggest that a number of the Bermuda grass allergens are still unknown [24], [25].

Given that the first contact between pollen grain and airway epithelium is with the pollen coat (surface) upon inhalation, pollen coat proteins may play a role in the genesis of allergic responses. In this study, we aimed to isolate, identify, and purify pollen coat proteins that bind IgE and examine their biochemical and immunologic properties.

## Materials and Methods

### Ethics Approval

All necessary permits were obtained for the described field studies, which were carried out in the Morton Arboretum of the University of Illinois-Urbana-Champaign (Lisle, IL, USA). Bermuda grass flowers were collected from the University of Illinois at Urbana-Champaign Turf Grass Research Farm and stored at −70°C until used. Dr. George Ware and Pat Apanavicius, the Morton Arboretum Secretary of Collections and Grounds Department, issued the permission for all of our field studies. These field studies did not involve endangered or protected species.

### Materials

#### Pollen

Defatted and raw un-defatted pollen grains: *Cynodon dactylon* (Bermuda grass), *Sorghum halepense* (Johnson grass), and *Phleum pratense* (Timothy grass) were purchased from Greer Laboratories (Lenoir, NC, USA) and stored with desiccant in tightly sealed containers at −20°C until used.

### Sera from Allergic and Non-allergic Individuals

#### Ethics statement

This research was approved by the University of Chicago Institutional Review Board, and documentation of this approval is available from the IRB office under protocol #15100B. The requirement for informed consent was waived because the study involved a retrospective chart review and screen of already collected sera. Sera from patients were de-identified and data were analyzed anonymously. Sera of allergic patients with clinically diagnosed allergy to grass pollen and a high titer of specific-IgE antibodies to specific pollen allergens by radioallergosorbent test (RAST) and ImmunoCAP were obtained from the University of Chicago’s Allergy Program in the Section of Otolaryngology-Head and Neck Surgery. Sera from individuals with negative allergen-specific antibody test results to grass pollen were used as negative controls. A pool of sera amassed from 500 individuals was purchased from Bioreclamation Inc. (East Meadow, NY, USA). The individuals were selected at random, and the pool contained an equal number of males and females. Pooled sera were partitioned into 1 ml aliquots and stored at −20°C until used.

## Methods

### Transmission Electron Microscopy (TEM)

For analysis in greater detail of the structure of the pollen coat after washing with organic solvent, TEM was undertaken on raw un-defatted, cyclohexane-washed, and commercially defatted BGP grains. For TEM analysis, the pollen grains were infiltrated by the use of vacuum and fixed, and then the monolayer of pollen was exposed to Acrolein vapor for seven days at room temperature in the dark. Samples were dehydrated in acid dimethoxypropane and acetone and embedded in Spurr resin (Electron Microscopy Sciences, Hatfield, PA, USA). Ultrathin sections were stained with saturated uranyl acetate and lead citrate and examined with a transmission electron microscope, Tecnai F30 (FEI Company, Hillsboro, OR, USA).

### Assessment of the Effects of Organic Solvents on Pollen Hydration

In preliminary studies, the potential of 24 organic solvents to extract pollen coat material without affecting the hydration of the pollen was evaluated. Pollen from Bermuda grass were put separately into tubes and extracted with protic and aprotic solvents that ranged from non-polar to polar. Each treatment tube was vortexed for 5 min after addition of the solvent. Aliquots of 100 µL from each solvent and pollen were examined by use of an inverted microscope. Bright-field images encompassing at least 100 pollen grains were photographed at 100× magnification for each treatment reaction after 30 min, 21 h and 7 days. Pollen was classified based upon specific morphologic characteristics. Nonhydrated pollen grains (NHPs) were classified by their oblong morphology, which resembles a deflated football, as opposed to round hydrated pollen. The isolated hydrophobic proteins from BGP were reconstituted in PBS, and protein concentrations were determined by the use of a BCA kit (Pierce, Rockford, IL, USA) according to the manufacturer’s protocol. After 30 min, 21 h and 7 days of treatment, pollen grains were categorized into three classifications. The first class consisted of hydrated pollen. The second class consisted of grains that were partially hydrated. The third class was NHPs. Pollen hydration and protein concentration assisted in identifying the solvents that extract enough pollen hydrophobic proteins without jeopardizing the integrity of the pollen surface or result in pollen hydration. On the basis of these factors, cyclohexane was found to be by far the best solvent for its high protein extractability and low pollen hydration rates (data not shown).

### Fractionation of Pollen Proteins into Coat and Cytoplasmic Proteins Based on Differential Solubility in Cyclohexane

Pollen coats (surfaces) from multiple allergenic species were extracted with use of cyclohexane as described previously [15], [16], [26]. Briefly, raw un-defatted pollen was vortexed in cyclohexane for 5 min. The pollen grains were separated from the solvent by vacuum filtration through a 3 µm mesh. The cyclohexane-washed pollen grains were retained for cytoplasmic protein isolation. The cyclohexane in the flow-through was evaporated under a stream of air, and the remaining material was resuspended in TBS-T (20 mM Tris, 136 mM NaCl, 0.1% Tween 20, pH 7.5). Proteins were precipitated with 80% ice-cold acetone and incubated at −20°C for 20 min. The precipitate was pelleted by centrifugation at 10,000×g for 10 min at 4°C. The supernatant was discarded and the pellet retained. Lipids remaining in the pellet were removed by first re-suspending of the pellet in 25 mM Tris, pH 7.5, 0.2% SDS. Trichloroacetic acid was added to a final concentration of 20%. The suspension was vortexed for 1 h at 4°C. The protein was precipitated by centrifugation for 10 min at 10,000×g and 4°C. The supernatant was discarded, and the pellet was washed three times with 100% acetone and allowed to dry at 4°C. The pellet was then re-suspended in 25 mM Tris, pH 7.5, 0.2% SDS and stored at −20°C. Protein samples destined for 2D PAGE were delipidated by the chloroform method described by Wessel and Flugge [27].

For isolation of the pollen cytoplasmic protein, the cyclohexane-washed pollen (from the previous step) was hydrated by suspension of the washed pollen in TBS-T (20 mM Tris, 136 mM NaCl, 0.1% Tween 20, pH 7.5) and mechanically ground with sea sand with a mortar and pestle and centrifuged at 3,000×g for 5 min at 4°C. The pellet was discarded, and supernatant was filtered through a glass filter. The cytoplasmic proteins were precipitated by 80% acetone. The mixture was vortexed briefly, incubated for 30 min at −20°C, and centrifuged for 10 min at 10,000×g and 4°C. The supernatant was discarded, and the pellet was resuspended in 25 mM Tris, pH 7.5, 0.2% SDS, and 10% glycerol. A cocktail of plant protease inhibitors (leupeptin, phenylmethanesulfonyl fluoride (PMSF), and E-64) was added to protein extracts at a 1∶100 dilution prior to storage at −20°C. Proteins were quantified with a Micro BCA Protein Assay Kit (Pierce Biotechnology, Rockford, IL, USA) according to the manufacturer’s instructions by use of a BioTek Synergy HT Multi-Mode microplate reader.

### SDS-PAGE and IgE-Immunoblot Analysis of Pollen Coat and Cytoplasmic Proteins

Purified BGP coat proteins were analyzed by one-dimensional SDS-PAGE with use of precast 12% Tris-glycine gels (Invitrogen, Carlsbad, CA, USA). Samples were electrophoresed for 90 min at 125 V. Proteins were visualized by either Coomassie blue staining or by use of Imperial Protein Stain Reagent (Pierce, Rockford, IL, USA) according to the manufacturer’s protocol. Two-dimensional electrophoresis was performed with use of Novex IEF gels (Invitrogen, Carlsbad, CA, USA), pH 3–10, for separation of proteins by isoelectric point, followed by separation by molecular weight on 4–20% polyacrylamide gradient gels on NuPAGE systems (Invitrogen, Carlsbad, CA), according to the manufacturers’ protocols.

For immunoblot analysis, proteins were electroblotted onto a 0.45 µm PVDF membrane (Millipore, Billerica, MA, USA) at 30 V for 90 min. After transfer, blots were blocked with a solution containing Tris-NaCl and 3% BSA. All membrane blots were incubated with a serum pool from patients with documented pollen allergy (1∶5 dilutions) or individuals with high IgE titers (University of Chicago, Allergy Program samples, 1∶7 dilution) in a solution containing Tris-NaCl and 1% BSA for 16 h at 4°C. Primary antibody was detected with horseradish peroxidase-conjugated monoclonal anti-human IgE (clone GE-1, Sigma; 1∶500 dilution), and the bands were revealed by addition of a SuperSignal® West Pico Chemiluminescence Substrate (Pierce, Rockford, IL, USA) following the manufacturer’s instructions.

### Electroelution of Cysteine Protease from Stained Gels

Raw un-defatted pollen surface extract was separated by SDS-PAGE. The protein bands were visualized by rinsing of the gel in ddH_2_O for 30 s, incubation in 0.2 M imidazole containing 0.1% SDS for 15 minutes, and de-staining in 0.1 M zinc sulfate. We used the Electro-Eluter from Bio-Rad to purify IgE-reactive CP and EXY from the BGP coat. The SDS-PAGE gel bands of the CP and EXY were excised with a scalpel. The proteins were electrophoretically eluted from the minced gel as previously described [28] and precipitated by use of the GeBAflex-tube system (Gene Bio-Application Ltd, Kfar Hanagide, Israel). Purified proteins were resuspended in 0.1 N NaOH and separated by SDS-PAGE to confirm their purity. CP and EXY were used in antigen-specific ELISAs to determine the frequency of IgE-binding to these proteins.

### Proteomic Analysis of Pollen Coat Proteins and Protein Sequencing and Identification

A proteomic approach was employed for identifying and analyzing the novel pollen-coat proteins from BGP. Pollen-coat proteins from Timothy and Johnson grass were also analyzed. Briefly, protein bands were excised and de-stained and sent to the Donald Danforth Plant Science Center Proteomics and Mass Spectrometry Facility (St. Louis, MO, USA) for all peptide sequencing and fingerprinting. After trypsin digestion and purification with HPLC, peptides were analyzed by a Voyager-DE STR MALDI-TOF mass spectrometer (Applied Biosystems, Foster City, CA, USA). The protein sequencing was performed with an Applied Biosystems ‘Procise’ 494 HT machine for direct sequencing of proteins and peptides by the established technique of N-terminal protein sequencing or Edman degradation. The peptide mass fingerprint data were searched against public sequence databases and user-supplied sequences for identification of peptides as identical to, or showing extensive homology with, known protein sequences (p<0.05) by use of a MASCOT v1.9 database-searching engine.

### Cloning and Sequencing of cDNA from Bermuda Grass Floral RNA Encoding the Cysteine Protease

Bermuda grass genomic DNA fragments were amplified via PCR by use of primers designed from highly conserved regions of corn and rice cDNA sequences. Corn and rice cDNA sequences were chosen from the Mass-Spectrometry Peptide Identification matched proteins. PCR products were cloned with use of a TOPO TA kit (Invitrogen, Carlsbad, CA) and sequenced with a 3730XL genetic analyzer (Applied Biosystems). We used the Bermuda grass cDNA sequence to design primers for the Rapid Amplification of cDNA Ends (RACE). Total RNA was extracted with the RNeasy® Plant Mini kit (QIAGEN, Valencia, CA, USA), and cDNA was synthesized from total RNA by use of the SMART™ RACE cDNA amplification kit (CLONTECH, Mountain View, CA, USA).

### Proteinase Activity Assay with Fluorescein Casein Used as Substrate

Proteinase activity in pollen coat proteins of Bermuda, Johnson, and Timothy grass was quantitated by use of fluorescence-conjugated casein (Molecular Probes, Invitrogen, Carlsbad, CA, USA) as substrate. The casein substrate (10 mg) was diluted in 50 mM Tris (pH 7.5), 150 mM NaCl, 5 mM CaCl_2_, and 0.01% NaN_3_ and incubated at room temperature for 16 h with 100 µl pollen extract. The digested substrate has absorption/emission maxima at 495 nm/515 nm, and its fluorescence intensity was measured with use of a Synergy HT Multi-Mode Microplate reader for detecting quantitative differences in activity.

### Gelatin and Casein Zymography Analysis

Proteases present in the pollen surface extracts were detected by their capacity to degrade gelatin or casein according to standard protocols [29]. Briefly, 10 µl (20 µg/lane) of pollen surface proteins, equalized for protein concentration (2 mg/mL), were added to non-denaturing loading buffer and subjected to electrophoresis on Novex 10% Zymogram SDS-PAGE with 0.1% gelatin or 0.05% casein as substrates incorporated into the gel. After electrophoresis and washing twice with 2.5% (v/v) Triton X-100, the gels were incubated overnight at 37°C, and immersed in a developing buffer (50 mmol/L TRIS-hydrochloric acid, pH 7.4, supplemented with 1% Triton X-100, 5 mmol/L calcium chloride, 10^–6^ mol/L zinc chloride, and 0.02% sodium azide). Afterwards, the gels were stained with 0.25% Coomassie brilliant blue R-250 for 1 h, and de-stained to expose proteolytic bands in 50% methanol and 10% acetic acid for 1 h. Pre-stained molecular-weight markers (Bio-Rad, Hercules, CA, USA) were included for estimating the molecular weight of the proteinase activity bands. The proteinase activity was evidenced as clear bands (zones of gelatin or casein degradation) against the blue background of stained gelatin and casein.

### Human Airway Epithelial Cell Monolayers

The airway epithelial cell line A549 (ATCC) was cultured as described previously [30]. Cells were seeded in sterile 24-well culture plates (Costar) in Ham’s F-12 medium supplemented with 10% heat-inactivated fetal calf serum (FCS) and 0.1% penicillin-streptomycin solution and grown to 80% to 90% confluence [30].

### Detachment Assay

A549 cells were seeded at 1×10^5^ cells/well on 24-well plates and incubated for 24 hr at 37°C. Cell monolayers were then treated with Bermuda pollen coat cysteine protease at concentrations of 0, 1, 3, 10, 30, 100, and 300 nM for 0 to 3 h. After protease treatment, cells were gently washed with 1×PBS during shaking for 10 min for removal of loosely attached cells. Adherent cells were fixed, dyed with crystal violet (0,75% crystal violet, 50% ethanol, 0.25% NaCl, 1.75% formaldehyde), and incubated at room temperature for 30 min as described previously [31]. Cells were washed twice with PBS and air-dried. Cells were examined with a Zeiss Axiovert 40 CFL inverted light microscope with a high NA 10X objective, and microscopic photos were taken with the Zeiss AxioCam ICc1 digital camera system. After microscopy, cells were lysed in elution solution (1% SDS in 1×PBS) overnight. The staining intensity was measured in a BioTek Synergy HT Multi-Mode Microplate Reader at 620 nm. The percentage of detached cells was expressed relative to that of untreated control cells (mean ± SD of three wells from four independent assays).

### *In vitro* Permeability Assays

Paracellular permeability was studied by measurement of the apical-to-basolateral flux of fluorescein isothiocyanate (FITC)-dextran (FITC-dextran 40 kDa, FITC-dextran 70 kDa, Sigma, St Louis, MO, USA) and IgG-horseradish peroxidase (HRP) (150 kDa, Southern Biotechnology) through confluent A549 airway epithelial monolayers as described previously [31], with modifications. A549 cells were seeded onto a 6.5 mm Transwell Collagen-coated 0.4 µm pore PTFE membrane insert at 5×10^4^ cells/insert in 200 µl of Ham’s F-12 medium supplemented with 10% heat-inactivated FCS. The lower compartment was filled with 800 µl of the same medium. A549 monolayers were grown for 6 days to confluence. For measurement of paracellular flux, 40 kDa or 70 kDa of FITC-dextrans or IgG-HRP-150 kDa were dissolved in PBS buffer, pH 7.2. The apical surface of the cell monolayers were treated with a solution of 0, 1, 3, 10, 30, 100, or 300 nM purified cysteine protease for 0 to 3 h. After the indicated time points of the cysteine protease treatment, transwell inserts were replaced for measurement of paracellular flux. Cells were allowed to equilibrate in PBS buffer for 10 min, and FITC-dextrans (40 kDa, 70 kDa, or IgG-HRP-150 kDa) were added to the apical compartment to give a final concentration of 1 mg ml^−1^. The basolateral medium was collected every 30 min, and the concentrations of FITC-dextrans were measured with a fluorometer by use of a BioTek Synergy HT Multi-Mode microplate reader (excitation 485 nm, emission 535 nm). Non-treated epithelial cells served as a negative control, whereas trypsin-treated cells served as a positive control for tight-junction disruption.

### Cell Stimulation and Cytokine Measurements

The airway epithelial cell cultures were starved for 16 h prior to the commencement of each experiment. Monolayers were stimulated with pollen coat preparations or pollen coat purified proteins in serum-free medium, and incubated at 37°C in a 5% CO_2_ atmosphere. Incubation with serum-free medium served as a negative control (basal cytokine secretion). After 24 hours of incubation with the pollen surface extracts, the cell supernatants were collected and stored at –70°C. Each experiment was performed in triplicate. IL-6, IL-8, IL-10, IL-13, IL-17 and TNF-α cytokines were assayed with use of commercially available ELISA kits (R & D Systems, Minneapolis, MN, USA).

### Polymorphonuclear Leukocyte (PMN) Transmigration Assay

The polymorphonuclear leukocyte (PMN) transmigration assay on inverted cell culture monolayers of polarized cells has been described previously [32]. Briefly, inverted monolayers were washed and equilibrated in Hank’s Balanced Salt Solution (HBSS) for 30 min. Inverted A549 monolayers were then flipped over and treated with 25 µl of pollen coat proteins. After 1 h, all monolayers were washed and flipped back into 24-well plates. For a positive control of the ability of PMNs to migrate, 10^−8^ µM fMLP was added to the bottom (apical) chamber of the untreated monolayers for each experiment. PMNs (1×10^6^) were then added to the top (basolateral) chamber, and the plate was placed at 37°C for 2 h. PMN that fully migrated into the apical chamber were quantified by myeloperoxidase assay [32]. PMN transmigration was expressed as mean ± SD of at least three independent assays.

### Measurement of Allergen-Specific IgE Antibodies in Human Serum by ELISA

Pollen grass sera were screened by ELISA for specific IgE to BGP coat CP or EXY as previously described [16]. In brief, a Nunc Immuno MaxiSorp 96-well plate (NUNC, Rochester, NY, USA) was coated with BGP coat protein extract or purified pollen coat cysteine protease or endoxylanase at a concentration of 1 µg/mL in PBS, and wells reserved for standards were coated with anti-human IgE (KPL, Gaithersburg, MD, USA) at a concentration of 2 µg/mL in PBS. After washing with PBS-0.05% Tween 20, and blocking with 10% fetal bovine serum (FBS) in PBS, a standard serial dilution of purified human IgE, from 20 to 0 ng/mL (Fitzgerald, Concord, MA, USA), was added to wells reserved for standards. The rest of the plate was incubated with serum samples from patients allergic to pollen grass (1∶5 dilutions in 10% FBS in PBS) and incubated overnight at 4°C. Plates were then washed, followed by incubation with mouse anti-human IgE conjugated to HRP (Serotec, Raleigh, NC) at a concentration of 1 µg/mL for 1 h at 25°C. After washing, SureBlue TMB/Peroxidase Substrate (KPL, Gaithersburg, MD, USA) was added according to the manufacturer’s protocol, and the reaction was stopped with TMB stop solution (KPL, Gaithersburg, MD, USA). Absorbance was read at 450 nm with a BioTek Synergy HT Multi-Mode microplate reader, and the amount of antibody binding was extrapolated from the standard calibration curves.

### Mouse Model of Allergic Rhinitis

Pathogen-free, female C57Bl/6 wild-type mice, aged 8–10 weeks, were purchased from Jackson Laboratory (Bar Harbor, Maine, USA). All mice were housed in environmentally controlled, specific pathogen-free conditions for a 1-week acclimatization period and throughout the duration of the studies. All animal care and experimental procedures were performed in accordance with NIH and USDA guidelines, and approved by the University of Chicago Institutional Animal Care and Use Committee (Protocol number: 72–164).

### Intranasal Sensitization of Mice by the Cysteine Protease of Bermuda Grass Pollen (CP-BGP)

The CP-BGP was purified as described previously. CP-BGP was used in all experiments. Mice (n = 5/treatment) were inoculated with 15 µl of 10 µg CP-BGP intranasally (i.n.) as 7–8 drops of aqueous solution into each nostril by microsyringe (Hamilton Co, Reno, NV, USA) on 5 consecutive days/week for three weeks. From day 28, mice were challenged with 15 µl of 10 µg on 7 consecutive days in the same fashion. This will be referred to as the nasal challenge.

### Analysis of Eosinophils by Flow Cytometry

Twenty-four hours after the last i.n. challenge, mice were euthanized. The naso-pharyngeal associated lymphoid tissue (NALT) was harvested, and the total number of cells in each mouse was determined. The cellular composition of the NALT was determined by differential flow cytometry (FACS) staining by use of the type-specific cell surface markers for eosinophils CD45+/CCR3+/B220−/CD3-. For staining, all conjugated monoclonal antibodies specific for mouse antigens were purchased from eBioscience, Inc. (San Diego, CA, USA) except anti-mouse CCR3 phycoerythrin (PE) from R&D Systems Inc. (Minneapolis, MN, USA). The following reagents from eBioscience were used for flow cytometry: Anti-CD3e APC (clone 145-2C11) for CD3+ T cells, anti-CD45R/B220-Pe-Cy7 or Pe-Cy5 clone RA3-6B2 for B lymphocytes, and anti-CD45 APC-Cy7 (clone30-F11) for leukocyte common antigen. The appropriate isotype controls were used for identification of background staining. Flow-cytometric analysis was performed immediately after staining. Data acquisition and analysis were performed on a BD LSR III flow cytometer (eBioscience, Inc., San Diego, CA, USA) with BD FACSDiva software and FlowJo (TreeStar, Cosa Mesa, CA, USA) software.

### Measurement of Allergen-Specific and Total IgE Antibodies in Mouse Serum by ELISA

Allergen-specific IgE concentrations were determined by direct ELISA, whereas total IgE was determined by sandwich ELISA as described previously [33].

## Results

### Organic Solvent Extraction of Pollen Grains Alters the Pollen Coat (Surface) Structure

**Figure 1:**
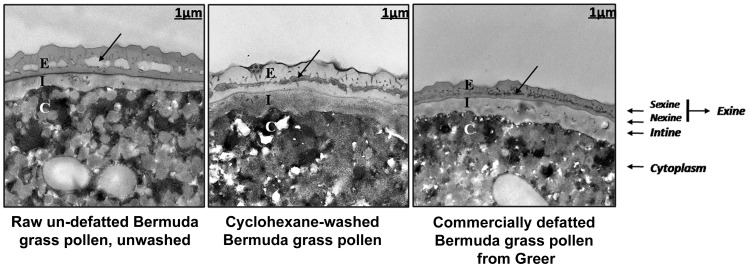
Transmission electron micrographs (TEMs) of BGP after extraction with organic solvents. The microchannels of the raw un-defatted pollen grain are covered with electron-dense surface coat materials, whereas in defatted pollen the microchannels of the surface coat are almost empty of electron-dense material. The microchannels of the exine layer, indicated by arrows, appear to collapse after organic-solvent extraction. The close proximity of the exines’ dark-staining inner channel surfaces makes the collapsed channels of the washed pollen darker than those of the raw un-defatted pollen. The supplier washes un-defatted pollen with n-Hexane to produce commercially defatted pollen. E, exine; I, intine; C, cytoplasm.

The organic solvent cyclohexane strips the lipids and proteins of the pollen coat from *Arabidopsis thaliana* without causing the pollen grain to hydrate or release its cytoplasmic contents [15]. Because the pollen grains used for preparation of commercial standardized allergenic extracts are defatted by extraction with organic solvent (typically petroleum ether, acetone, or hexane), we wanted to determine whether the surface layers are altered by this treatment. The results of transmission electron microscopy (TEM) confirmed that cyclohexane extraction or commercial delipidation of pollen had significantly altered the pollen surface (Figure 1). Figure 1 indicates that the microchannels of the raw un-defatted BGP grain were covered with electron-dense surface coat materials, whereas in defatted pollen the microchannels of the surface coat were almost empty of electron-dense material. The microchannels of the exine layer appeared to collapse after organic solvent extraction or delipidation. The close proximity of the exines’ dark-staining inner channel surfaces made the collapsed channels of the washed pollen darker than those of the raw, un-defatted pollen. This alteration may be due to the loss of pollen coat proteins and lipids and the subsequent collapse of surface channels.

### **P**ollen Coats from Bermuda, Timothy, and Johnson Grass Contain IgE-reactive Proteins

**Figure 2:**
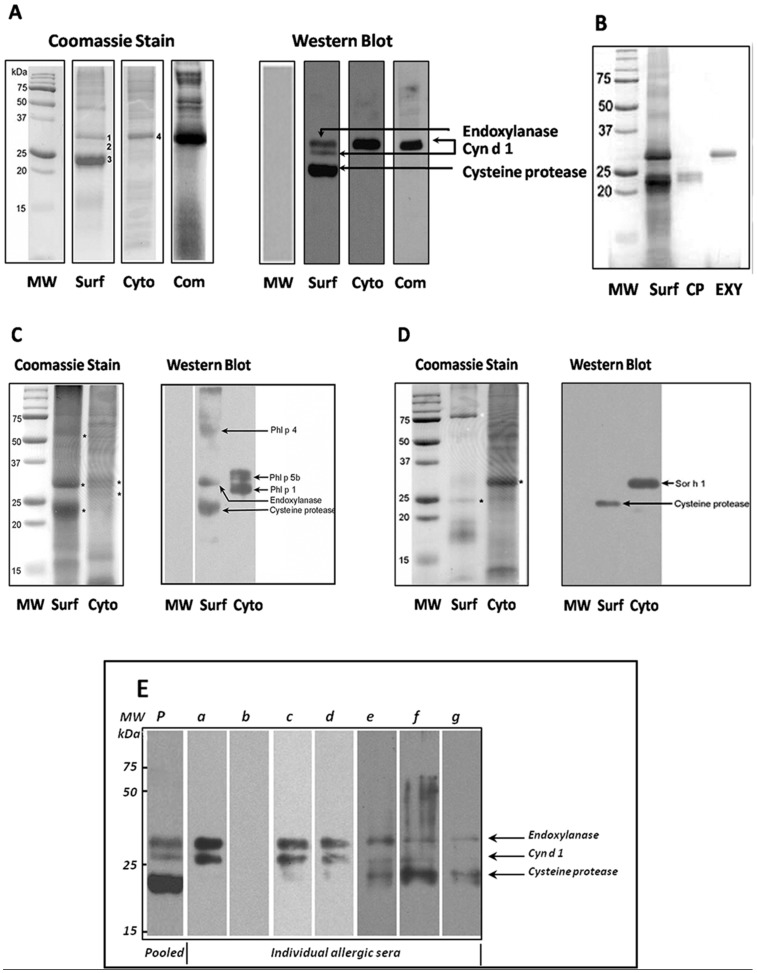
SDS-PAGE and immunoblotting analyses of in-lab preparations of pollen surface (coat), cytoplasmic proteins and commercial defatted pollen from Bermuda grass, Timothy grass, and Johnson grass. Raw un-defatted pollens were extracted with cyclohexane. Cyclohexane-soluble proteins (pollen coat) were separated, and the remaining cyclohexane-defatted pollen was used for isolation of the cytoplasmic fraction. MW markers, surface pollen coat and cytoplasmic proteins, and commercial defatted Bermuda grass pollen (Greer) were resolved by (**A**) SDS-PAGE and stained with Coomassie blue (*left panel*) or transferred to nitrocellulose membranes, probed with human pooled allergic sera, and detected with anti-human IgE antibodies (*right panel*) as described in Materials and Methods. Three numbered protein bands, 1, 2, and 3, from the Bermuda pollen surface, denoted IgE-reactive proteins identified in the immunoblot, were sequenced and identified via MALDI-TOF-MS and peptide sequencing as endoxylanase (EXY), major allergen Cyn d 1, and cysteine protease (CP). The BGP coat proteins were resolved by SDS-PAGE. Proteins identified as IgE-reactive CP and EXY were cut from the gel and electro-eluted for high purification of each protein (**B**). The pollen surfaces of (**C**) Timothy and (**D**) Johnson grass pollens also contain IgE-reactive CP. Three protein bands from the Timothy pollen surface, indicated by stars, were sequenced and identified as major allergen Phl p 4, EXY, and CP. A CP was identified from the Johnson grass surface. The data shown are taken from one representative experiment repeated three times. (**E**) Frequency and identity of IgE-reactivity of Bermuda pollen surface proteins. Proteins were analyzed by Western blotting for the presence of IgE and tested with pooled (*p*) and individual (*a–g*) pollen-allergic patients’ sera. Each lane corresponds to an allergic patient serum. The 23 kDa band was identified as CP. The data shown are taken from 1 representative experiment repeated three times.

Because the cyclohexane-soluble fraction of BGP is enriched in pollen coat (surface) proteins, the term “cyclohexane-soluble proteins” or “pollen coat proteins” will be used interchangeably in this report to indicate the pollen proteins on the surface of pollen extracted by cyclohexane. For study of the BGP coat proteins following cyclohexane extraction, the BGP-coat proteins (cyclohexane-soluble) were separated by SDS-PAGE in parallel with proteins isolated from the cytoplasm of washed pollen grains. Figure 2A shows that the pollen-coat fraction contains a number of proteins that are not present in the cytoplasmic fraction. The SDS-PAGE of pollen coat proteins and the cytoplasmic proteins of Bermuda grass revealed an abundance of protein components in the 15, 25, and 50 kDa ranges, whereas the cytoplasmic fraction contains few proteins of these sizes. This suggests that the pollen coat and cytoplasmic proteins differ in identity and abundance.

Because there are several distinct extractable proteins in these two pollen fractions, we wanted to study whether any of the pollen coat proteins showed reactivity toward IgE from grass-pollen-allergic patient sera by using Western blot analysis. To that end, surface and cytoplasmic proteins from BGP were resolved by SDS-PAGE and stained with Coomassie blue (Figure 2A, left panel), transferred to nitrocellulose membranes, probed with human pooled allergic sera, and detected with anti-human IgE antibodies. Western blot analysis revealed the presence of three intense IgE-reactive protein bands in the pollen-coat fraction and one band from the cytoplasmic extract (Figure 2A, right panel), indicating the presence of cross-reactive antigenic determinants in the two pollen extracts.

### Proteomic Analysis of Pollen Coat Bands from One Dimensional SDS-PAGE

**Figure 3:**
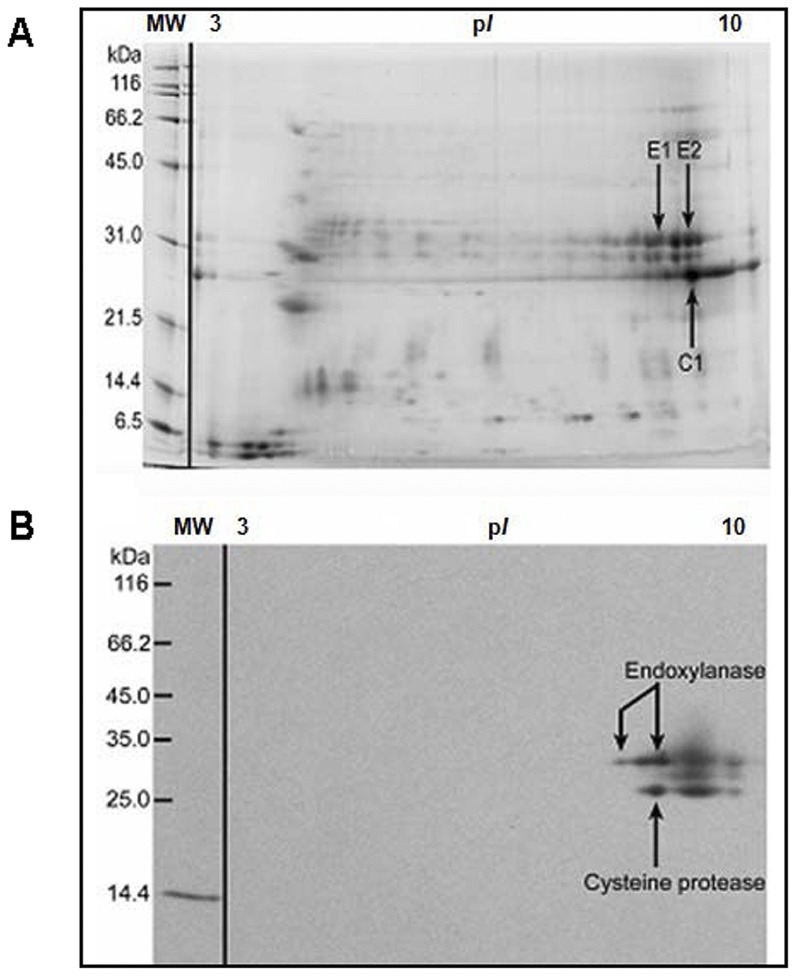
2D-SDS-PAGE of BGP coat proteins (cyclohexane-soluble) stained with Sypro Ruby and IgE-immunoblotting. Pollen coat proteins were separated first by isoelectric point and then by molecular weight. Labeled spots were excised, destained, and digested with trypsin for peptide mass fingerprinting. (**A**) The identities of spots E1, E2, and C1 were verified by mass-spectrum peptide identification as two endoxylanases and a cysteine protease. Two-dimensional IgE-immunoblotting of BGP coat proteins (**B**). Approximately 50 µg of pollen coat proteins were loaded into an IPG strip (pH 3–10). Following 2D-SDS-PAGE, Western blotting was performed, and the membranes were probed with human pooled allergic sera and detected with anti-human IgE antibodies as shown in Figure 2. The IgE-reactive spots corresponded to E1, E2, and C1 spots on 2D-SDS-PAGE in Figure A. IgE in the pooled sera also reacted with the 14.4 kDa mass marker, lysozyme from chicken egg white. 2D-SDS-PAGE and 2D-Western blot were repeated three times and generally found to be reproducible.

We undertook a proteomic approach to identify and analyze the gel-separated protein bands at molecular weight (MW) 23 kDa and 30 kDa from the BGP coat. The bands were excised from the gel, de-stained, digested with trypsin, and then analyzed by use of MALDI-TOF-peptide Mass finger printing at the Donald Danforth Plant Science Center, Saint Louis, MO. The peptide mass fingerprints from MALDI-TOF-MS analysis of the two bands corresponding to the IgE-reactive bands in Western blot and the proteolytically active bands in the gelatin zymogram of the BGP coat identified two novel IgE-binding proteins: an endoxylanase (EXY) and cysteine protease (CP), from the cyclohexane-soluble fraction. The last IgE-reactive protein in the cyclohexane-soluble fraction and the cytoplasmic fraction was identified as the major BGP allergen, Cyn d 1. To confirm that only one protein was migrating with the endoxylanase or cysteine protease, we performed 2-dimensional gel electrophoresis (2-DE) and 2D IgE-immunoblots with the pollen coat fraction (Figures 3A and 3B). The 2-DE maps were established for the endoxylanase and cysteine protease. The different protein spots in the 2-DE were analyzed with image analysis software; and then the peptide mass finger printing of the different proteins was verified by MALDI-TOF mass spectrometry. The identities of the two IgE-reactive protein spots thought to be isoforms of endoxylanase (E1 and E2 spots) and one spot believed to be a cysteine protease (C1 spot) were verified and confirmed through Edman N-terminal sequencing and MALDI-TOF MS fingerprinting by searching of peptide masses against the Swissprot database and user-supplied sequences.

To confirm the identity of these IgE-reactive proteins further, we used N-terminal sequencing of CP and EXY for the design of PCR primers from corn and rice consensus sequences to amplify Bermuda grass CP and EXY via PCR. The mass-spectrum data were searched against BLAST (Basic Local Alignment Search Tool) databases for hits to a new amplified sequence. The protein sequence homologies between PCR amplified cysteine protease or endoxylanase and corn cDNA were ∼ 85%.

Multiple Cyn d 1 isoallergens have previously been positively identified as different isoforms of BGP, which have been reported to migrate at different sizes in SDS-PAGE [34]. Each isoallergen may show unique antigenic reactivity. It is likely that sequence and size variations indicate that different isoforms of the protein are present in each of the pollen surface and cytoplasmic fractions [34].

**Figure 4:**
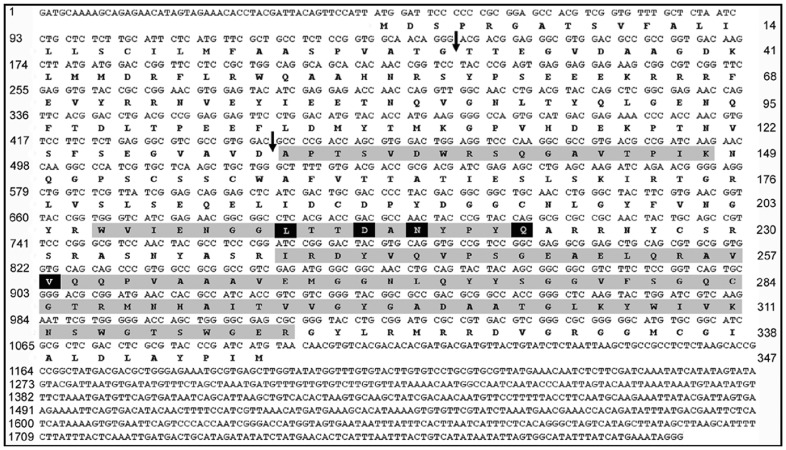
Cloning of the full-length cDNA and protein sequence of the BGP coat, IgE-reactive cysteine protease. The full-length cDNA sequence was cloned from Bermuda grass floral RNA. The protein sequence appears below the mRNA open reading frame. The numbers on the left indicate the nucleotide position at the beginning of each line. The numbers on the right indicate the amino-acid position at the end of each line. The arrows indicate the putative cleavage site for the signal peptide (top arrow) and the cleavage site of the pro-domain (bottom arrow). Gray shading indicates IgE-reactive band peptide sequence obtained from peptide sequencing by Edman degradation that matches the cDNA sequence. Sequences that do not match peptide-sequencing results are shaded in black.

To explore whether pollen coat proteins of other grass species may contain similar allergens, we resolved surface and cytoplasmic proteins from Timothy (*Phleum pratense*) and Johnson (*Sorghum halepense*) grass by SDS-PAGE and stained them with Coomassie blue (Figure 2C and 2D, left panels) or transferred them to a nitrocellulose membrane; we probed with human pooled allergic sera and detected with anti-human IgE antibodies (Figure 2C and 2D, right panels). Three protein bands from the Timothy pollen coat were sequenced and identified as the major allergen Phl p 4, endoxylanase, and cysteine protease. A cysteine protease was identified from the Johnson grass surface. Peptide sequencing of IgE-reactive bands confirmed that pollen coat proteins from Timothy and Johnson grass contained CPs of approximately the same molecular weight as that of the BGP pollen CP (Figure 2A, 2C, and 2D). The peptide-sequencing results suggest that Johnson and Timothy grass CPs are very similar to the protein encoded by the BGP-coat cysteine protease cDNA (Figure 4). Additionally, a pollen coat IgE-reactive endoxylanase from Timothy grass with a molecular weight similar to that of BGP endoxylanase was identified. A 30 kDa IgE-reactive expansin-like protein from the cytoplasmic fraction of Johnson grass was identified (Figure 2D), which is likely to be the previously identified allergen, Sor h 1. In addition to Sor h 1 from Johnson grass, a number of previously characterized allergens from Timothy grass, namely, Phl p 4, Phl p 5b, and Phl p 1, were identified.

### Cloning and Sequencing of cDNA from Bermuda Grass Floral RNA Encoding Cysteine Protease

Pollen surface proteins are synthesized and assembled onto the pollen grain by the diploid tapetal cells of the developing flower [7], [8]. We used Bermuda grass floral RNA to clone the cysteine protease cDNA, because the RNAs that code for these proteins are likely to be present only in floral tissues. To amplify the putative genes from the cDNA, we designed degenerate primers by using the peptide sequences of the IgE-reactive cysteine protease and from conserved sequences of similar cysteine proteases from *Zea mays* (corn) and *Oryza sativa* (rice). These degenerate primers were designed to minimize codon degeneracy, which increases the likelihood that they will be specific to the gene that encodes the candidate allergen. The primers produced a PCR product of approximately 300 bp (data not shown). This PCR product was sequenced and used for the design of primers for 5`- and 3`-Rapid Amplification of cDNA Ends (RACE), which was used for cloning of the full-length cDNA from the floral cDNA library. The presence of this cDNA in the Bermuda grass floral library suggests that this IgE-reactive protein is present on the surface of the pollen grain.

The full-length cDNA cloned is 1806 bp, and the largest open reading frame is 1044 bp, which encodes a 347 amino-acid protein (Figure 4). The first 30 amino acids of the protein are predicted by SignalP 3.0 software to be a signal peptide. Additionally, N-terminal sequencing showed that the protein contains a pro-domain of 101 amino acids (Figure 4). Once the pro-domain is cleaved (after amino acid 131), the mature protein consists of 216 amino acids with a calculated molecular weight of 23.6 kDa (Figure 4). This is the approximate size of the native protein when it is separated by SDS-PAGE (Figure 2A and 2B).

The cloned cDNA encodes a protein that significantly matches the peptide sequencing results of the native IgE-binding CP generated by mass spectrometry and Edman degradation sequencing (grey shade in Figure 4). Peptide sequencing of the native protein covered 54% of the mature protein derived from the full-length cDNA. NCBI-BLASTp analysis confirmed that the cloned cDNA has 96% identity to the peptide sequencing results (Figure 4) and shows significant homology to known CPs from maize and rice (data not shown). Although peptide-sequencing results significantly match the cloned protein sequence, there are discrepancies between the two sequences (Figure 4). In some cases, these discrepancies are the result of inconclusive peptide-sequencing results. In other cases, these discrepancies may indicate the presence of multiple isoforms of the CP. Indeed, the 2D gel results suggest that multiple isoforms of this CP do exist (Figure 3A).

Although the full-length cDNA of the BGP coat CP was obtained, attempts to express the recombinant Bermuda pollen coat CP in *E. coli* were not successful. Consequently, we used Baculovirus expression vectors to enable the expression of the recombinant CP in insect cells, a strategy that was also not successful.

### Analysis of Pollen Coat Cysteine Proteases

**Figure 5:**
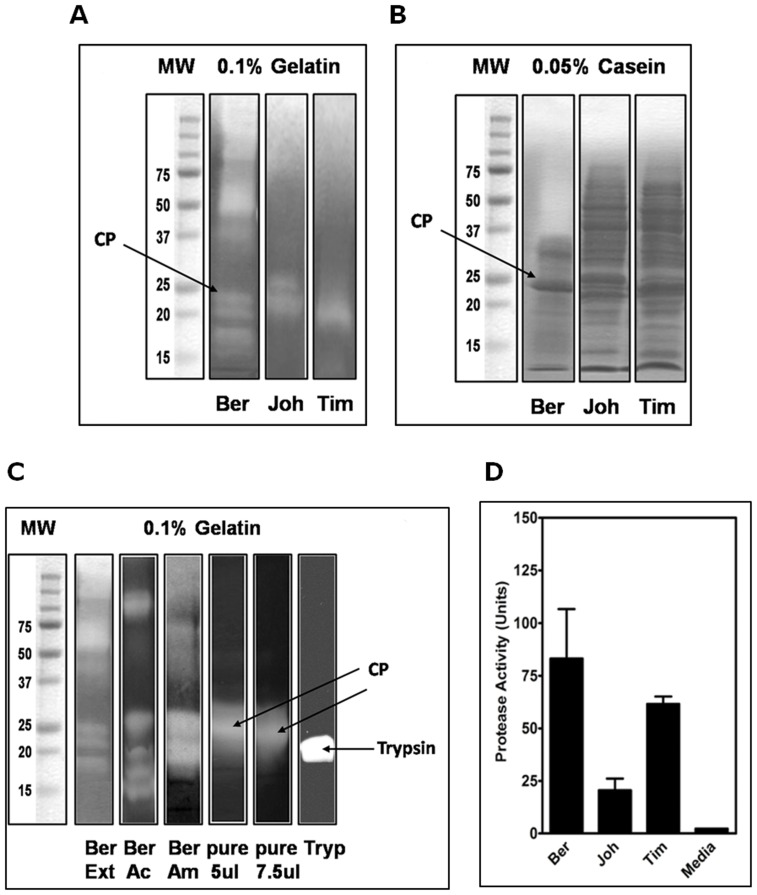
Total proteinase activity of pollen coat proteins from Bermuda, Timothy, and Johnson grass assessed by gelatin and casein zymography. Bands with proteinase activity were visualized as white clear or light lytic zones against a dark background of Coomassie-blue-stained gel (**A, gelatin**) and (**B, casein**). On the left, molecular mass markers in kDa are indicated. (**C**) Pollen coat proteins from Bermuda grass. MW markers were on the left lane, and crude extracts (Ext), concentrated with 80% Acetone (Ac) and with 70% ammonium sulfate (Am), pure cysteine protease (pure, 5 and 7.5 µg/lane) and trypsin as a positive control are shown. The gelatin zymography gives a representative image from 4 separate studies that yielded similar results. (**D**) Protease activities in pollen surface proteins from Bermuda, Johnson, and Timothy grass corresponding to the lanes in (A) and (B) in the upper panels. The protease activities of pollen surface proteins, equalized for protein concentration, were examined for proteinase activity by use of a chromogenic substrate. The activity was recorded and expressed as units of enzyme activity per milligram of total protein. The cysteine protease colorimetric assay shows significantly greater proteinase activity in the proteins extracted from the Bermuda grass pollen surface compared with proteins extracted from Johnson grass or Timothy grass pollen surface. Bars represent the mean of proteinase activity units of four replicates.

Proteinase activities in the pollen coat of Bermuda, Timothy, and Johnson grass were shown to digest a casein fluorescent substrate. However, at a similar protein concentration, Bermuda proteinases exhibited a high rate of proteolysis, followed by Timothy and then Johnson (Figure 5D). To complement the results of the fluorescent protease assay and to estimate the molecular weight of the associated proteinase, we further analyzed the BGP coat extract and the cysteine protease gel-purified by gelatin zymography. Zymography analysis demonstrated five intense proteolytic bands that have gelatin-cleaving activities and that corresponded to the 16–18 kDa, 23 kDa, and 75 kDa proteases from BGP coat proteins. The presence of the proteolytic enzymes varied among pollen species; however, the 23 kDa band seems to be dominant in the three types of pollen grass. The major proteolytic activities in the Bermuda pollen coat corresponded to the 23 kDa band. Western blots confirmed the existence of a band with a similar molecular weight that reacts to IgE in sera from human patients with a high titer of specific-IgE antibodies to pollen grass as documented by CAP/RAST, ELISA, and clinical history.

Our data are consistent with a single 23 kDa band that coincided with a strong Coomassie-blue-stained band on SDS-PAGE, an IgE-reactive band on Western blots, and a major proteolytic-activity band in a gelatin zymogram, potentially confirming the presence of a dual-function allergenic cysteine protease in the BGP coat. Mass spectrometry identification, peptide sequencing, N-terminal sequencing, and cloning of the full-length cDNA further confirmed the existence of the BGP coat CP.

### Pollen Coat Cysteine Protease-induced Rounding and Detachment of Airway Epithelial Cells

**Figure 6:**
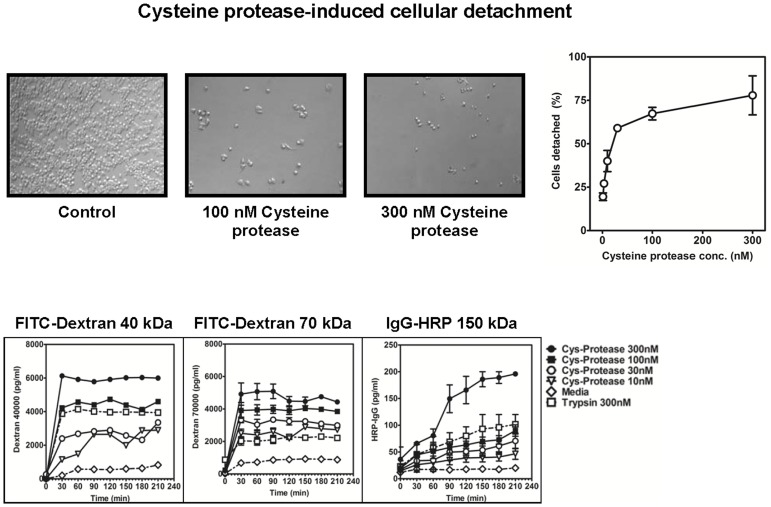
Concentration-dependent detachment of airway epithelial cells by pollen cysteine protease. Confluent monolayers of A549 epithelial cells were treated with Bermuda pollen coat cysteine protease for a 3-hour period *(upper left panels)*. After light microscopic observation, cell detachment was assessed as described in Materials and Methods. Light-microscopic photos of the average cell density of the epithelial cell exposed to the serum-free medium F-12 (0 nM) and to serum-free media containing (300 nM) cysteine protease are shown. After 1 hour of incubation, the cells were analyzed by light-microscopic imaging. Microscopic images were taken at ×10 magnification. Bar = 25 um. Each image is representative of three individual wells. Following microscopy, the percentage of detached cells was determined and is expressed as a percentage relative to detachment observed in untreated control cells *(upper right panel).* Error bars represent means ± SD of four independent experiments. Pollen coat cysteine protease increases paracellular permeability of A549 epithelial cells *(lower panel).* The apical surface of the confluent A549 airway epithelial monolayers were left untreated (0) or treated with a solution of 1, 3, 10, 30, 100 or 300 nM purified cysteine protease for the indicated time. Paracellular permeability of epithelial cells by measurement of the apical-to-basolateral flux of FITC-dextran 40 kDa, FITC-dextran 70 kDa and IgG-HRP 150 kDa as described in Materials and Methods are shown.

External proteolytic enzymes can directly cause damage to the airway epithelial cells and degrade components of the extracellular matrix of the cells. Airway damage is a feature of allergic rhinitis. House-dust mite (HDM) allergens with cysteine and serine proteinase activities have been reported to increase the airway epithelial permeability, thereby facilitating the access of allergens and allergic sensitization. On the other hand, the capacity of pollen coat proteases directly to induce damage to the human airway has not previously been studied. To test our hypothesis that the BGP coat CP proteolytic activity is critical for the dysfunction of the airway epithelial barrier, we exposed airway epithelial cell (A549) monolayers grown on glass slide matrix to the purified BGP coat CP. First, the capacity of BGP coat CP to induce epithelial cell detachment was evaluated visually and by crystal-violet staining. The results showed that the extent of epithelial cell detachment varied according to the time and concentration of the proteolytic enzyme. As shown in Figure 6, the cells that were incubated with CP showed a high percentage of detachment, whereas no cell detachment could be observed after incubation with medium. BGP-coat CP induced detachment of airway epithelial cells in a dose-dependent fashion; producing from 25% up to 70% detachment compared to the use of medium alone. The effect of the most concentrated CP was quite severe, with a high percentage of cell detachment from the monolayers.

Our observations indicate that the pollen coat releases a set of proteolytic proteins, and that among them is the cysteine protease that is directly able to induce epithelial-cell detachment and promote the disruption of the integrity of airway epithelial cells. These results are similar to those obtained when epithelial cells were treated with HDM *Dermatophagoides pteronyssinus* (Der p 1), a major allergen [35].

Transport of allergenic proteins across the airway epithelial barrier is a critical event in allergic sensitization. Here, we modeled the epithelial barrier *in vitro* by using a human airway epithelial-cell (A549) monolayer seeded on a Transwell membrane that served as a support. The diffusion of different-sized fluorescein isothiocyanate (FITC)-labeled dextrans and IgG-HRP was measured as a permeability index. For evaluation of the potential of the BGP coat CP to alter the permeability of the airway epithelial-cell monolayers, this model made it possible to test the barrier function of the treated cells. The results showed that CP treatment caused increased permeability of human airway epithelial cells, which did not occur when the cells were treated with medium alone. CP influences a broad spectrum of cellular functions, including cell detachment and changes in epithelial permeability, leading to barrier dysfunction.

### Cytokines Secreted by A549 Cells Stimulated with Pollen Coat Proteins

**Figure 7:**
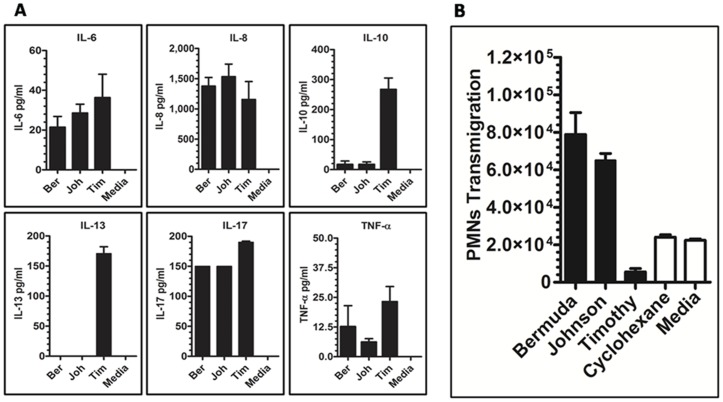
Stimulation of human airway epithelial cells with pollen coat proteins generates the pro-inflammatory cytokines IL-6, IL-8, IL-10, IL-13, IL-17, and TNF-α. Confluent airway epithelial cells were stimulated with Bermuda, Johnson, or Timothy pollen coat proteins (25 µg/ml) for 24 h, and cytokine levels in the culture media were measured by ELISA (**A**). Each bar represents mean ± SEM. Data are representative of 3 separate experiments. Significantly increased IL-8 levels were present in pollen surface protein-treated samples compared with PBS-treated samples (P≤0.05). Pollen coat protein PMN transmigration across airway epithelial cells (**B**). A549 monolayers were exposed to 25 µg pollen coat proteins, and the number of PMNs that had completely migrated across the epithelium into the apical chamber were quantified and presented as number of PMNs multiplied by 10^4^. HBSS medium represents untreated monolayers. Data represent mean ± SEM of three experiments each performed in triplicate.

Because the pollen coat is the first to encounter the mucosal immune system upon inhalation, and previous screens for pollen allergens have focused on cytoplasm proteins, this experiment focused particularly on the role of pollen coat proteins in the immune response. Using ELISA, we evaluated the secretion of cytokines for IL-6, IL-8, TNF-α, IL-13, IL-10, and IL-17 by human alveolar epithelial cells A549 24 h after stimulation with Bermuda (Ber), Johnson (Joh), or Timothy (Tim) grass. Timothy-stimulated A549 cells secreted higher levels of IL-6, IL-10 and IL-13 compared to the case for A549 cells cultured in the presence of Bermuda or Johnson or medium alone (Figure 7). In contrast, IL-10 and IL-13 were not were not detected. The results demonstrated that pollen surface proteins differed in their potency to induce inflammatory markers, and the order of potency among the proteins was different according to the specificity of the components present.

### Frequency of IgE Binding of Cysteine Protease and Endoxylanase from the BGP Coat among Allergic Patients

A direct ELISA to determine the prevalence of IgE-reactivity in sera from pollen-grass allergic patients to EXY and CP from the BGP coat was developed as described previously [16]. Sera from allergic patients with a high titer of specific-IgE antibodies to pollen grasses as determined by clinical history and RAST at the University of Chicago were used (Table 1). Sera from individuals with negative allergen-specific antibody test results to grass pollen have been used as negative controls. Representative results from selected pollen-grass-allergic patients are shown in Table 2. The majority of the selected patients showed a specific IgE reaction with both the CP and the EXY, indicating the presence of allergenic epitopes in pollen coat proteins. Allergen-specific ELISA using sera from individuals with negative allergen-specific antibodies to grasses did not show any signal or significant binding with BGP coat proteins, BGP-CP or BGP-EXY (not shown).

**Table 1:** Percentage of allergic individuals with IgE to the BGP coat fraction.

	Percentage
**All allergic individuals^#^**	25
**High-IgE individuals^♦^**	57

* All allergic individuals had a positive RAST result to Timothy grass pollen. # n = 36.

**♦**High-IgE individuals with RAST value >2000 kU/L to Timothy grass pollen. n = 14.

**Table 2:** Percentage of allergic individuals with IgE-reactivity to the BGP coat cysteine protease and endoxylanase.

	Percentage
**Endoxylanase**	75
**Cysteine Protease**	63

All allergic individuals had a positive ELISA result to Bermuda grass pollen coat fraction. n = 8.

Allergen-specific ELISA using sera from individuals with negative allergen-specific antibodies to grasses did not show any signal or significant binding with BGP coat proteins, BGP-CP or BGP-EXY (not shown).

Of the individuals with positive radioallergosorbent test (RAST) and CAP results to grass pollen, 25% had IgE binding to the BGP coat fraction. Of those individuals with grass-pollen RAST values greater than 2000 kU/L, 57% had IgE reactivity to the BGP coat fraction. Of the individuals who were reactive to the BGP coat fraction (Table 1), 63% and 75% were IgE-reactive to the CP and EXY, respectively. These results suggest that the ELISA results shown in Table 1 were not skewed by the presence of Cyn d 1 in the pollen coat fractions. Additionally, these data suggest that a significant number of patients diagnosed with allergy to grass pollen showed IgE reactivity to the BGP coat proteins EXY and CP (Table 1).

### Allergenicity of CP-BGP in a Murine Model of Allergic Rhinitis

**Figure 8:**
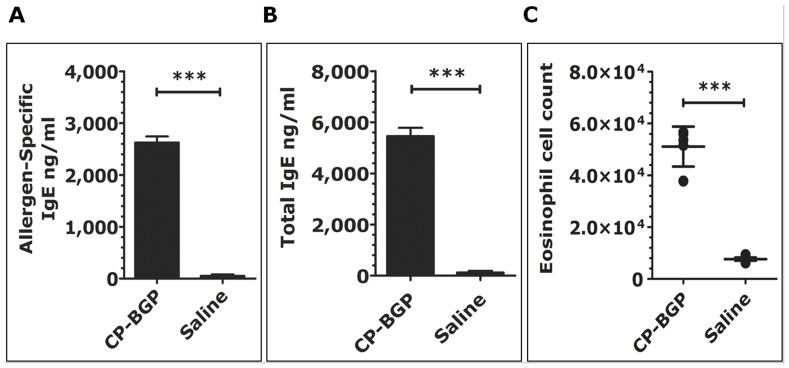
Intranasal administration of CP BPG enhances the allergen-specific IgE and total IgE Abs response. Four weeks after the initial intranasal sensitization mice were bled. Allergen-specific IgE (**A**), total IgE (**B**) levels in sera from CP-BGP or saline C57BL/6 mice were measured by ELISA. Individual serum samples were measured and expressed as the geometric means ± SEM (n = 5 per group). (**C**) Increased eosinophil influx into nasal mucosa of C57BL/6 mice sensitized intranasally with CP-BGP. Cell counts were determined by differential FACS staining of cells isolated from the nasal mucosa. Data is shown as mean ± SD (n = 5 per group). Significant differences (*** p<0.001 compared with paired control group. One representative experiment of two is shown.

Having demonstrated that CP-BGP was capable of stimulating A549 epithelial cells and inducing pro-inflammatory cytokines, we next tested the potential of CP-BGP to induce an IgE allergic response in a murine model. Figure 8 shows that mice sensitized intranasally with CP-BGP had significantly higher allergen-specific IgE, total IgE antibodies, and eosinophil influx into the nasal mucosa compared to control groups (p≤0.001, all). The results presented indicate that, upon i.n. sensitization with CP-BGP, high allergen-specific IgE and total IgE levels are produced, without the use of adjuvant. These data suggest that the dual function of CP-BGP preferentially results in T_H_2 cell activity leading to an enhanced-IgE antibody response. Based on these results, it seems reasonable to suggest that the dual functional effects of the CP-BGP present in the same molecule are essential for its potent allergenicity.

## Discussion

Bermuda grass pollen (BGP) is one of the most potent and clinically important allergens of the grasses of the *Poaceae* family [36], [37], and several IgE-reactive allergens have been identified in this family [17], [20], [21], [22], [23], [38], [39], [40]. The traditional searches for pollen allergens have used defatted pollen and extraction methods that favor the isolation of cytoplasmic proteins [17], but have overlooked many of the pollen coat proteins. In this study, we showed that defatted BGP, washed with the organic solvent cyclohexane, has an altered surface layer compared to raw, un-defatted pollen. A previous report has shown that washing un-defatted *Arabidopsis thaliana* pollen with cyclohexane removes the surface proteins from the pollen grain without hydrating the pollen or releasing the cytoplasmic contents of the pollen grain [15].

In this study, we showed that the pollen coat fraction from BGP contains IgE-reactive proteins that have not been identified previously. Peptide-sequencing results suggest that the novel IgE-reactive proteins with molecular weights of 30 kDa and 23 kDa are homologous to EXYs and CPs from maize and rice. These proteins were identified by their reactivity toward IgE from the sera of grass pollen-allergic patients. The results showed that a significant number of sera from individuals with pollen allergy contain IgE to BGP coat proteins, indicating the importance of proteinases from the pollen coat in the allergic response.

Although numerous searches for BGP allergens have been performed, the starting material used has been defatted pollen. These searches using defatted pollen have identified a number of IgE-binding cytoplasmic proteins, including the group I allergen, an expansin called Cyn d 1; a calcium-binding protein, Cyn d 7; a profiline, Cyn d 12; a pathogenesis-related protein, Cyn d 24; a flavoprotein, BG60; and a cytochrome c oxidase, Cyn d Bd46k [20], [21], [22], [23], [38], [39], [40]. Interestingly, in a previous study, Kao and colleagues, who used a proteomic approach to identify novel allergens from defatted BGP, did not identify the IgE-reactive bands at the sizes and pI (isoelectronic point) of the endoxylanase or cysteine protease [15], suggesting that these allergens are not present in defatted BGP.

Because these IgE-reactive proteins are not present in defatted pollen and were isolated only from raw, un-defatted pollen extracted with cyclohexane, they are likely to be pollen surface (coat) proteins. Pollen surface proteins are synthesized and assembled onto the pollen grain by the tapetal cells of the maternal flower [6], suggesting that the genes which encode these proteins are expressed during floral development. Therefore, we used Bermuda grass floral RNA for cloning the full-length cDNA that encodes the pollen coat, IgE-reactive CP.

A full-length cDNA that encodes a protein with significant homology to the papain-like C1 family of CPs was cloned [41]. Interestingly, the closest homologue in Arabidopsis (At1g06260) is expressed in the immature inflorescences according to the Massively Parallel Signature Sequencing database, suggesting that this protein is also pollen surface-specific [42]. The cloned cysteine protease significantly matches the sequences generated from peptide sequencing of the IgE-immunoreactive band, suggesting that the correct cDNA was cloned.

Attempts efficiently to express the CP enzyme in common expression hosts had failed. As yet, we have no explanation as to why the expression construct vector of recombinant CP enzyme had failed to express the protein properly in *E. coli* or insect cells. This raises the possibility that the expression construct could not utilize the full protein-processing machinery of the expression host cell, or failed to yield a properly folded recombinant domain. Intriguingly, the intrinsic toxicity of nucleases, as well as the potential cytotoxicity and proteolytic activity, have been suggested as causes of the failure to express proteolytic enzymes in expression host cells. Another cause of expression failure could be the divergences in codon and nucleotide composition from the host gene.

In addition to the Bermuda grass cysteine protease, we identified similar pollen coat CPs from other grass species, including Timothy and Johnson grass. The size of the CP is approximately 23 kDa, and partial peptide sequencing demonstrates that portions of these proteins are very similar to the BGP coat CP. To date, IgE-reactive CPs have not been identified in either Timothy or Johnson grass (for a review/list of known grass allergens, see ref [43]). These proteins were identified by use of the same, pooled, and individual allergic patient sera that were used for identifying the Bermuda grass CP.

Additionally, ELISA results showed that almost 70% of patients allergic to grass pollen display IgE reactivity to BGP-coat proteins. Taken together, these results suggest that there is cross-reactivity between these pollen-coat CPs. A number of grass allergens are cross-reactive, including the well-characterized group I allergens [27], [44], [45], [46], [47]. The group I allergens are a family of ß-expansins, to which about 90% of grass pollen allergic patients display IgE reactivity [43].

These ß-expansins are glycoproteins that range in size from 31 to 35 kDa, and that include Cyn d 1, Sor h 1, and Phl p 1, which were IgE-reactive in our cytoplasmic fractions. Interestingly, Cyn d 1 was also identified in the pollen coat fraction, although the protein was slightly smaller compared to the cytoplasmic Cyn d 1. This suggests that these are different isoforms, which have been reported to have different sizes in SDS-PAGE [34].

Pollen coat components can cause release of inflammatory cytokines in the human alveolar epithelium. The strategic location of proteases on the allergenic pollen surface and their close proximity to other pollen allergens could contribute to the pro-allergic milieu that is characteristic of allergic sensitization or exacerbation in tissues exposed to pollen grains. These findings strengthen the hypothesis that pollen coats contain allergenic components with different potencies that induce an inflammatory response in human alveolar epithelial cells.

The CP-BGP proteolytic activity seems substantially to increase both the immunogenicity and the allergenicity of proteins. Taken together, our results support the notion that CP-BGP possesses a multidimensional capacity to stimulate the immune system and indicate that a major mechanism of its allergenicity is associated with the IgE-binding capacity, the ability to stimulate B cells, and the proteinase activity.

In this work, a group of pollen-coat, IgE-reactive proteins were identified, which have not been identified in previous searches for allergens. These include a group of CPs, which have been identified in all grass species tested thus far, and a group of EXYs, which have been identified in two of the three species tested. These proteins are likely to be pollen-surface proteins, because the solvent used to purify them from the pollen has been shown to strip only surface proteins from the pollen without hydrating the pollen and without causing the release of the cytoplasmic contents of the pollen grain [15], [16]. Organic solvents like cyclohexane are used for removal of lipids from the pollen surface before standardized allergenic extracts are prepared as required by the FDA to control the level of potency and narrow the range of variability from one lot to the other [14]. Here, we showed that this process visually alters the pollen surface and also removes important IgE-binding proteins, suggesting that allergen extracts used for diagnosis via skin-prick tests or RAST probably lack these potentially important allergens.

Because these IgE-reactive proteins are removed from the pollen surface with an organic solvent like cyclohexane, they are likely to be non-polar molecules. Generally, allergens are thought to be polar molecules; however, we have shown that proteins which are present in a non-polar, lipid-rich environment are IgE-reactive. This has important implications for allergen sources other than pollen, including indoor and food allergens, where the focus has been on the identification and characterization of polar allergens. The field of non-polar allergens has been largely unexplored, and we illustrated that, at least with pollen, there are numerous non-polar IgE-reactive proteins.

In this paper, we reported the identification, cloning, and characterization of a 23-kDa novel dual-function allergen from Bermuda grass located on the pollen coat that acts as a proteolytic enzyme. The first step in allergy treatment is to identify the agent that triggers the allergy. Currently, allergy sufferers are usually diagnosed or treated with pollen extracts, which may or may not contain proteins from the pollen coat. The unique approach we took to identify this allergenic protein from the pollen coat (surface) may lead to the identification and development of allergy-diagnostic reagents for screening. The identification of additional pollen coat allergens does more than just add to the catalogue of proteins that cause allergy-related symptoms; it provides the potential for additional diagnostic and treatment options for allergy sufferers.

Our results would suggest that some epitopes responsible for antigen-specific IgE antibody production (allergic epitopes) may be present on the pollen coat. The identification of additional allergenic pollen coat proteins and epitopes needs to be undertaken for the development of potential immunotherapy based on pollen coat allergenic epitopes.

Because the pollen coat is the first structure of the pollen grain to come into contact with the mucosa of the upper respiratory tract, the presence of protease on the pollen would probably be very important in the allergic response. BGP-coat CP may become useful as a diagnostic reagent and as a model allergen for the study of early events of allergic sensitization. In further studies of the BGP-coat CP, their ability to promote sensitization via the respiratory tract could be evaluated in a bioassay, possibly involving intranasal exposure in animal models of allergic rhinitis.
